# Recognizing Lipofuscinosis as a Guide in Antiepileptic Treatment: Clinical Description of the First Mexican Case With Neuronal Ceroid Lipofuscinosis Type 7 (NCL7)

**DOI:** 10.7759/cureus.56914

**Published:** 2024-03-25

**Authors:** Tamara N Kimball, Andrea G García-Rueda, Pamela Rivero-García, Aarón H Pérez-Segovia, Luis E Mayoral-Carrasco

**Affiliations:** 1 Genetics, Instituto Nacional de Ciencias Médicas y Nutrición Salvador Zubirán, Mexico City, MEX; 2 Radiology, Instituto Nacional de Ciencias Médicas y Nutrición Salvador Zubirán, Mexico City, MEX; 3 Genetics, Instituto Nacional de Neurologia y Neurocirugia Manuel Velasco Suarez, Mexico City, MEX

**Keywords:** ncl7, pathogenic variant, seizures, pigmentary retinopathy, mfsd8, neuronal ceroid lipofuscinosis

## Abstract

Neuronal ceroid lipofuscinosis type 7 (NCL7) is a rare form of childhood dementia; it is part of a group of diseases characterized by rapid progressive cognitive decline, blindness associated with retinitis pigmentosa, and seizures. We report the clinical and molecular characteristics of the first Mexican patient with NCL7, highlighting a particularly atypical disease course. The typical presentation form is expected to have reduced life expectancy and an average age of ambulation loss at 12 years. Our 27-year-old patient retains the ability to walk. The patient's unique presentation could, in part, be attributed to her genetic profile: a hypomorphic allele carrying a missense variant (c.1390G>A) and an almost null allele with a frameshift variant (c.1086del), contributing to the preservation of some protein function. Throughout her childhood and early adulthood, our patient experienced a variable response to antiseizure drugs, attributed to a lack of recognition of the disease and the specific efficacy of certain antiseizure medications. Our findings underscore the significance of considering this genetic condition and acknowledging its clinical heterogeneity.

## Introduction

Neuronal ceroid lipofuscinoses (NCLs) are a group of clinically and genetically heterogeneous neurodegenerative diseases caused by pathogenic variants (PVs) in 13 genes. NCLs are among the most common causes of dementia in childhood with an estimated incidence of one per 100,000 individuals and a prevalence of two to four per 1,000,000 individuals [1]. They are characterized by intracellular accumulation of autofluorescent lipopigments. These deposits are found in neurons but are also abundant in non-neuronal cells outside the nervous system. Depending on the subtype, the storage material is either predominantly composed of subunit C of the mitochondrial ATP synthase (SCMAS) or the sphingolipid activator proteins A and D. Manifestations include intellectual disability, motor impairment, visual loss, and drug-resistant seizures, with progressive physical decline and early death. Historically, they are classified into four major subtypes based on the age of presentation and ultrastructural abnormalities found with electron microscopy: infantile, late infantile, juvenile, and adult [2]. To date, 13 NCL disorders have been characterized based on the affected gene and the clinical phenotype; all of them follow an autosomal recessive (AR) mode of inheritance with the exception of NCL type 4, which is inherited in an autosomal dominant form. NCL type 7 (NCL7, OMIM#:610951) is an AR inherited disorder caused by PVs in the *MFSD8* gene, which encodes for a transmembrane lysosomal protein mainly expressed in the brain and eyes antenatally [3]. Here, we report the case of a 27-year-old female patient with molecularly confirmed NCL7, which, to our knowledge, is the first in Mexico.

## Case presentation

**Figure 1:**
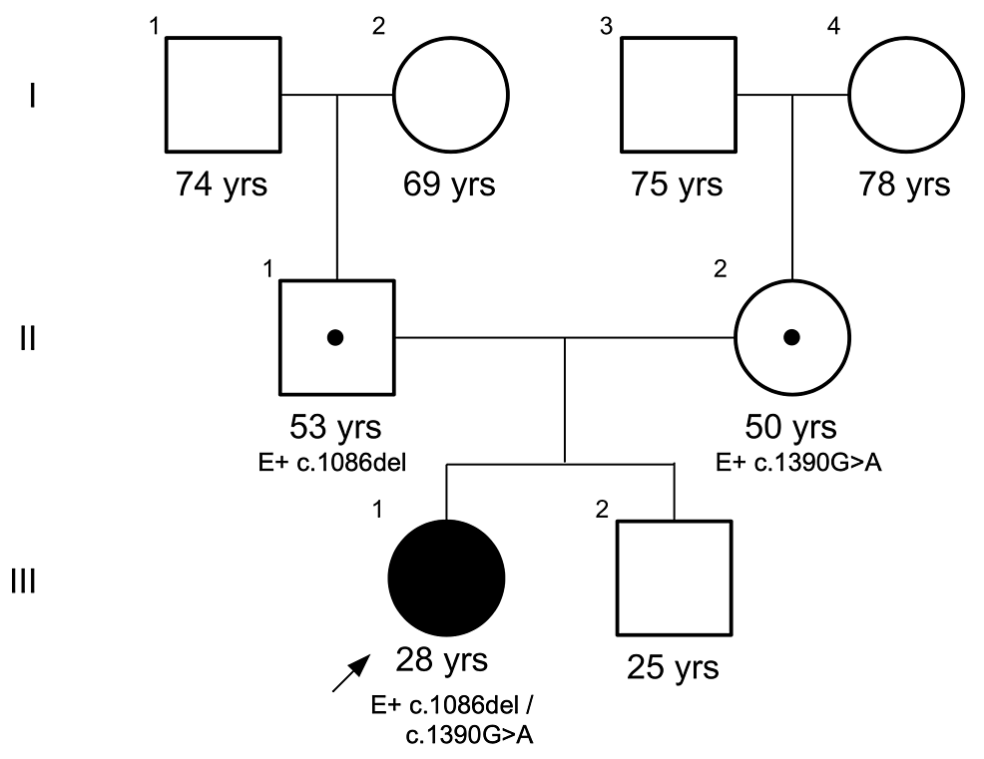
Family pedigree, which highlights the absence of consanguinity. Individual III.1 is our proband and is marked by an arrow in the pedigree.

The patient was born to non-consanguineous parents. Family history is unremarkable (Figure 1). She exhibited typical psychomotor development. At six years old, she debuted with seizures of focal onset (tonic seizures in lower extremities), which progressed to bilateral tonic-clonic seizures accompanied by impaired awareness. They occurred predominantly during sleep, without aura, lasting around two minutes. Valproic acid was initiated, presenting one episode of seizures every two months. Two years later, she developed progressive vision loss. A fundoscopy documented salt-and-pepper pigmentation and paleness in both papillae, compatible with pigmentary retinopathy (PR). Inborn errors of metabolism and mitochondriopathies were ruled out with complementary studies; an expanded metabolic screening, which included amino acids and acylcarnitines in the blood; and enzymatic activity of mitochondrial complexes in muscle biopsy. From age 11 to 15, treatment was initially changed to phenytoin and later to lamotrigine, with one seizure per month. At 15 years, management with levetiracetam and topiramate was started, with complete seizure control.

**Figure 2:**
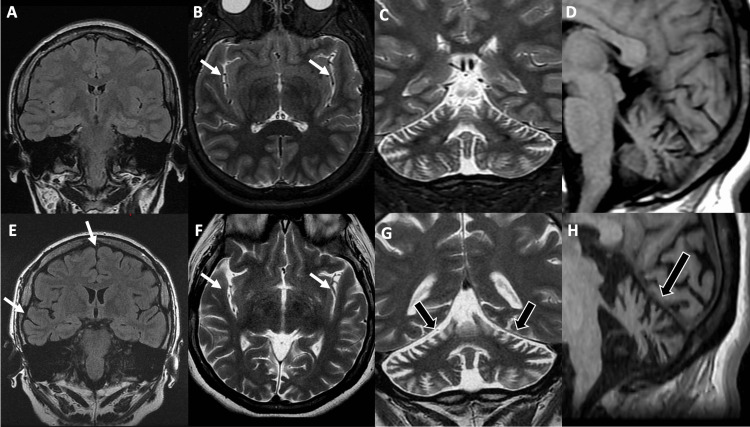
Brain MRI of a female patient with neuronal ceroid lipofuscinosis type 7 at 18 years (A-D superior) and 26 years old (E-H inferior) Comparative coronal fluid-attenuated inversion recovery (FLAIR) (A and E), short-TI inversion recovery (STIR) (B), and T2 (F) images show mild supratentorial volume loss with mild cortical atrophy (white arrows). Comparative coronal T2 (C and G) and T1 (D and H) show prominent folia of the cerebellar hemispheres, representing the progression of cerebellar atrophy (black arrows).

At 18 years, she was referred to our institution. Physical examination revealed complete blindness and horizontal gaze-evoked nystagmus. A brain MRI uncovered a slight increase in the sulci, prominent cerebellar fissures with incipient atrophy, and mild cortical atrophy (Figure 2A-2D). During the following years, she developed behavioral problems. After a five-year seizure-free period, antiepileptic treatment was suspended. However, six months later, she required emergency care due to uncontrolled seizures; the semiology of these seizures was similar to previous episodes, being generalized tonic clonic. Levetiracetam was initiated with seizures occurring every three months. At the age of 26 years, lacosamide was added, and the frequency of seizures increased to one every 15 days. A second brain MRI showed a more pronounced loss of volume of cerebellar hemispheres and vermis (Figure 2E-2H). 

Next-generation sequencing of 330 genes associated with pigmentary retinopathy was performed, revealing two compound heterozygous variants in the *MFSD8* gene: c.1086del (p.Ile364Tyrfs*50) and c.1390G>A (p.Ala464Thr). The first one, a frameshift variant, was classified as pathogenic using the American College of Medical Genetics and Genomics criteria [4]. The latter, originally classified as a variant of uncertain significance, was reclassified as likely pathogenic because of the highly compatible clinical manifestations of our patient and the allelic configuration in trans, corroborated by cascade testing on both parents. After establishing the diagnosis of NCL7, combination therapy with levetiracetam 1.5 g bid and topiramate 100 mg bid was initiated. The patient has not experienced any seizures since that time.

## Discussion

Previous cases of other types of NCLs have been reported in Mexico. Bravo-Oro et al. reported two cases of siblings with CLN6 variants, in a compound heterozygous state, causing NCL type 6 [5]. Furthermore, Kousi et al. described a patient with NCL type 8 and a CLN8 variant [6]. However, to our knowledge, this is the first report of a Mexican patient with NCL7 confirmed by molecular testing.

Due to its heterogeneity and overlapping symptoms, it is difficult to establish a differential diagnosis between NCLs; however, clinical manifestations and age of onset may help to guide toward a specific NCL type [1].

Our patient presented with partial seizures at age 6, which is similar to what has been previously described in patients with NCL7, ranging from 2 to 7 [7]. Life expectancy is around 12 years, although a few cases over 18 have been reported [8,9]. To date, at 27 years of age, our patient progression shows memory loss, dysarthria, and behavioral problems (irritability and emotional lability). Interestingly, she has adequate control of seizures and mild cognitive impairment and still conserves the ability to walk. This could be explained by the presence of one hypomorphic allele, carrying a missense variant (c.1390G>A) and an almost null allele, with a frameshift variant (c.1086del), therefore preserving some function of the protein. Likewise, different genetic and environmental factors could act as modifiers at different stages and affect the progression. Further research is essential to investigate these factors comprehensively and develop precise therapies capable of altering the course of the disease.

**Figure 3:**
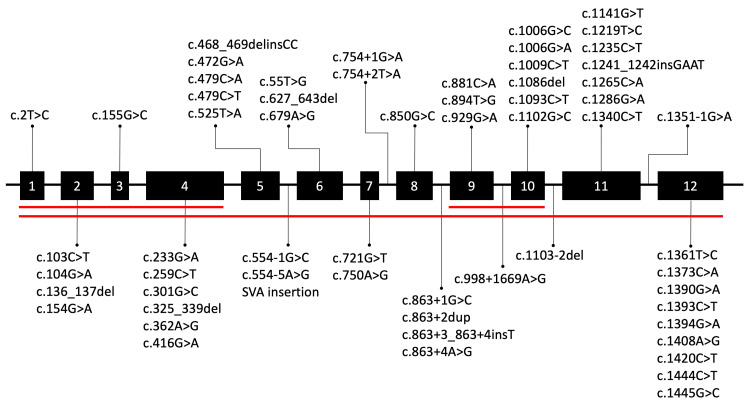
Distribution by location in the NCL7 gene of the pathogenic variants reported in the literature Genomic rearrangements, particularly deletions are denoted by the red lines. This figure is an original creation by the authors.

We reviewed all the cases of NCL7 reported in the literature. We found 63 different variants in *MFSD8* (Figure 3); 96.8% (61/63) are point mutations and three large deletions. Most point mutations are located in exon 12 (15%, 9/61), exon 11 (11.7%, 7/61), and exons 10 and 4 (10%, 6/61 each).

As demonstrated in this case, individuals with NCL7 can have a very effective response to certain anticonvulsants, such as valproic acid, lamotrigine, topiramate, and levetiracetam, contrary to carbamazepine, phenytoin, vigabatrin, and lacosamide, which have been associated to an increase in seizure activity [10].

## Conclusions

Prompt recognition of this entity is of the utmost importance. Although there is no curative treatment, it can guide management. Lack of control of the seizures is related to clinical deterioration and major burden on the patients and caregivers. This case highlights the clinical heterogeneity of NCL7 and emphasizes the importance of bearing in mind these genetic disorders, even in patients with syndromic epilepsy who reach adulthood without a definitive diagnosis. PR and seizures of infantile-onset accompanied or not by behavioral disturbances should raise suspicion of NCL7.
